# γ-Radiation Reduces phosphorylated-Tau in RhesusMacaque Brains: Potential Implications forAlzheimer’s Disease and other Tauopathies

**DOI:** 10.21203/rs.3.rs-9077029/v1

**Published:** 2026-04-02

**Authors:** Erin K. Murphy, Kathleen Hatch, Stephen Y. Wise, Oluseyi Fatanmi, Sarah A. Petrus, Manish Bhomia, Daniela Lecca, Barbara E.C. Knollmann-Ritschel, Daniel Perl, Vijay K. Singh, Diego Iacono

**Affiliations:** Uniformed Services University of the Health Sciences; Uniformed Services University of the Health Sciences; Uniformed Services University of the Health Sciences; Uniformed Services University of the Health Sciences; Uniformed Services University of the Health Sciences; Uniformed Services University of the Health Sciences; Uniformed Services University of the Health Sciences; Uniformed Services University of the Health Sciences; Uniformed Services University of the Health Sciences; Uniformed Services University of the Health Sciences; Uniformed Services University of the Health Sciences

**Keywords:** Alzheimer’s disease, γ-radiation, Neuro-radiotherapy, Tau radiosensitivity, Tauopathies, Radio-hormesis

## Abstract

**Background:**

Studies on the effects of γ-radiation on nonhuman primate (NHP) brains are limited, despite the critical need to understand the impact of radiation exposure on the brain from various sources like radiotherapy equipment, space travel, and potential nuclear events.

**Methods:**

We investigated molecular and neuropathological changes in rhesus macaque brains after a single 5.8 Gy total-body γ-radiation exposure. We analyzed samples dissected from frontal cortex (FCtx), hippocampus (Hippo), and cerebellum (CRB) of irradiated (RAD) vs. unirradiated/control (CTRL) animals. Western blotting and digital PCR (dPCR) analyses were used to measure different phosphorylated-Tau (pTau) forms and neurodegeneration markers (i.e., amyloid protein precursor [APP], neurofilament-light chain [NFL], glial fibrillary acidic protein [GFAP], ionized calcium-binding adapter molecule 1 [IBA1/AIF1], and myelin basic protein [MBP]).

**Results:**

We detected lower levels of different forms of soluble pTau species (pTau181, and pTau217, among others) in RAD vs. CTRL animals across all three examined brain regions. While APP and GFAP levels were unchanged in the FCtx, increased IBA1 and NFL levels were detected alongside decreased MBP levels. Moreover, dPCR data identified decreased expression of GFAP and MBP in the FCtx. Importantly, the molecular changes observed were not accompanied by overt signs of neurodegeneration or cellular abnormalities upon neuropathological assessment.

**Conclusions:**

These findings in irradiated NHPs’ brains are novel and indicate that a single total-body γ-radiation exposure significantly alters soluble pTau levels after a few weeks from irradiation without causing obvious neurohistological damage. These results open intriguing new possibilities of exploring γ-radiation-based strategies to modulate the progression of tauopathies, including Alzheimer’s disease.

## Introduction

Current knowledge regarding the specific biomolecular and neuropathological effects of γ-radiation on normal brain tissue in nonhuman primates (NHPs) is severely lacking, if not virtually absent [1]. This gap in research is profound, considering the urgent necessity of understanding these effects for several high-stakes domains. Indeed, a deeper comprehension of γ-radiation’s impact on NHP brains - the closest surrogate to human brains - is crucial for refining radiotherapy protocols for the treatment of brain tumors [2], developing effective countermeasures for accidental or deliberate radiation exposure (e.g., radiological accidents or nuclear warfare) [3, 4], and accurately evaluating the risks associated with occupational exposure and space travel [5–7], especially concerning Galactic Cosmic Rays (GCR) [14].

While rodents remain the dominant model in biomedical research, their neurobiological responses often fail to translate to humans, especially in the context of central nervous system (CNS) injury or pathologies. This sharply contrasts with NHPs, like the rhesus macaque, which offer a more translationally relevant system [8–10], and which mirror human-like physiological and pathological responses to various types of stressors, including irradiation.

The value of NHPs is already largely recognized by various research communities who have attempted to use NHPs to model neurodegenerative diseases such as Alzheimer’s disease (AD), Parkinson’s disease (PD), and amyotrophic lateral sclerosis (ALS) [11–15]. Nonetheless, the historical reluctance to conduct long-term NHP studies, primarily due to logistical and social challenges, is increasingly concerning given the limited success of curative neurodegenerative disease treatments following decades of rodent-based research [16, 17]. Moreover, the escalating risks from radiological threats [3, 4, 18], possible nuclear conflicts, and the advent of extended human space missions (e.g., Mars exploration) [19] critically underscore the need for comprehensive, ethically sound NHP studies that accurately reflect human neurophysiology and pathology [20]. Building on some recent findings in a minipig model that showed significant reductions in key AD-related proteins (Tau and phosphorylated-Tau [pTau]) [21] shortly after radiation exposure [22–24], we aimed to translate and expand these investigations into this even more relevant primate model [25–30].

In the present study, we systematically evaluated the possible neurodegenerative-associated molecular and neuropathological effects across various regions of captive-bred rhesus macaques following a few weeks after an acute total-body γ-radiation (5.8 Gy) exposure. Specifically, we measured the expression levels of multiple Tau species (HT7, pTau-S202-T205, pTau-T181, pTau-T217, pTau-S202, 3R-Tau, 4R-Tau), alongside other markers commonly associated with neurodegeneration such as amyloid precursor protein (APP), neurofilament light (NFL), myelin basic protein (MBP), glial fibrillary acidic protein (GFAP) and ionized calcium binding adaptor molecule 1 (IBA1). All these targeted markers were assessed in three anatomically and functionally distinct regions of the NHPs brain: the frontal cortex (FCtx), hippocampus (Hippo), and cerebellum (CRB).

## Methods

### Irradiated (RAD) Animals.

This study involved four clinically healthy rhesus NHPs (*Macaca mulatta*) between 3 to 7 years of age and weighing 4 to 7 kg (3 male, 1 female). These NHPs were housed and cared for at Southern Research (SR) in Frederick, MD. Upon arrival at SR’s facility, the animals completed a mandatory quarantine period of seven weeks. Animal housing rooms were maintained at a temperature of 22 °C±2 °C, the relative humidity range was kept between 30–70%, 10 to 15 air change cycles per h, and a standard 12-h light–12-h dark cycle. Animals were fed a certified diet (Purina LabDiet 5048) and received regular nutritional enrichment. Drinking water was provided *ad libitum*. The study design and all animal procedures were evaluated and approved by The Institutional Animal Care and Use Committee and the Department of War Animal Care and Use Review Office. All procedures involving the animals strictly adhered to the Guide for the Care and Use of Laboratory Animals and ARRIVE guidelines throughout this study as previously described [31,32].

### Unirradiated (Control/CTRL) Animals.

Control tissues were collected from unirradiated (CTRL) rhesus macaques as closely matched in age and sex to the irradiated (RAD) cohort as possible. Tissues from control animals were obtained from Southwest National Primate Research Center (SNPRC) at Texas Biomedical Research Institute, San Antonio, TX. SNPRC is accredited by AAALAC International. Upon arrival to Texas Biomedical Research Institute, animals complete a mandatory quarantine period of 30 days. Animal housing rooms were maintained at a temperature of 20–30 °C, the relative humidity range was kept between 30–70%, 10 to 15 air change cycles per h, and a standard 12-h light–12-h dark cycle. Animals were fed a certified diet (Purina LabDiet 5048) and received regular nutritional enrichment. Drinking water was provided *ad libitum*. All animal procedures were evaluated and approved by the Institutional Animal Care and Use Committee of the Texas Biomedical Research Institute. All procedures involving the animals strictly adhere to the Guide for the Care and Use of Laboratory Animals and the Public Health Service Policy on Humane Care and Use of Laboratory Animals.

### Radiation Exposure.

Animals were paired for radiation exposure, with pairings determined by similar abdominal width measurements (within ±1 cm) taken at the abdominal core using digital calipers. Radiation exposure to these animals occurred between 8:00AM and 12:00PM. Approximately 15 min before irradiation, animals were sedated with an intramuscular (*im*) injection of ketamine hydrochloride (10–15 mg/kg, 100 mg/ml). Once adequately sedated, animals were then secured in Plexiglas restraint boxes which were custom-made and secured in position using tethers attached to their limbs and a strap around their waist. Excessive limb movement after restraint was addressed with an additional 1.5–5.0 mg/kg *im* ketamine injection to prevent movement during the procedure. The two restraint boxes were positioned facing away from each other on the irradiation platform and were exposed to cobalt-60 total-body g-radiation at a dose of 5.8 Gy and a dose rate of 0.6 Gy/min. The dose of 5.8 Gy total-body irradiation (TBI) was selected because it is the expected dose that will cause 30% lethality in rhesus macaques within 60 days of exposure (LD_30/60_) [33,34]. Immediately following the radiation exposure, NHPs were placed back in their home cages and closely monitored by study staff until they fully recovered from sedation (able to stand or sit upright on their own). Additional details of the total-body irradiation procedure and dosimetry are described previously [35].

### Euthanasia.

The study period was intended to be 60 days, but two of the four animals were euthanized early due to moribundity to prevent unnecessary pain and suffering. This can be attributed to the specific radiation dose used (5.8 Gy TBI, LD_30/60_). All sample collections for the CTRL animals were obtained opportunistically as determined by veterinary staff at SNPRC and after excluding possible direct neurological pathologies as cause of death. Euthanasia was conducted in accordance with the American Veterinary Medical Association guidelines as previously described [36]. Briefly, RAD and CTRL animals were sedated with an *im* injection of ketamine hydrochloride (5–15 mg/kg), then euthanized by sodium pentobarbital intravenously (>100 mg/kg, Euthasol, Virbac AH, Inc, Fort Worth, TX). Absence of pulse, breathing, and heartbeat served as the criteria for confirming death.

### NHP Brain-necropsy.

At the time of necropsy, the skull cap was removed using an oscillating bone saw. The brain was dissected away from the skull. After gently extracting the brain from the skull, its weight was recorded and then it was placed on a flat surface to separate the two hemispheres (cerebral and cerebellar) and the brainstem. The left fresh hemi-brain was placed on a thick sheet of paper and then stored at −80 °C. The right hemi brain was placed in a solution of phosphate-buffered 4% paraformaldehyde fixative.

### Protein Extraction and Western Blot (WB).

Each left cerebral hemisphere was cut into 100 μm thick sections in a cryostat and further microdissected by anatomical regions, including but not limited to frontal cortex (FCtx), hippocampus (Hippo), and cerebellum (CRB). The dissections were guided by following rhesus monkey brain atlases [37, 38].

The collected brain regions were homogenized in glass dounce homogenizers with ice cold lysis buffer (1 ml/100 mg tissue; 50 mM Tris-HCl (pH 8), 1% Igepal, 150 mM NaCl, 1 mM EDTA, 1 mM PMSF, 1 mM NaF, 1:100 protease inhibitor cocktail (Sigma-Aldrich, P2714, St. Louis, MO). Samples were centrifuged at 12,000 × g for 20 min to separate the cytosolic (supernatant) and nuclear (pellet) fractions. Supernatants were collected, aliquoted and frozen at −80 °C. Total protein content for each brain region was determined using the Micro BCA assay (Thermo-Fisher Scientific, 23235, Waltham, MA, USA). A total of 20 μg of protein per sample, for all brain regions listed, were loaded on Novex Nupage 4–12% Bis-Tris Gels (Life Technologies, NP0329, Carlsbad, CA, USA) and were electrophoresed at 200 V constant for 30 min. Gels were transferred to PVDF membranes using the iBlot2 dry transfer method (Life Technologies, IB21001, Carlsbad, CA, USA). Membranes were blocked in 5% milk in 1X TBST for 1 h at room temperature (RT). The primary antibodies (see paragraph below) were diluted to the appropriate working concentrations in Intercept Protein-Free Antibody Diluent (Li-Cor, 927–85001, Lincoln, NE, USA) and incubated on the membranes overnight at 4 °C. Membranes were then rinsed 3x five min in TBST. Appropriate HRP tagged secondary antibodies (see paragraph below) were diluted 1:2000 in Intercept Protein-Free Antibody Diluent (Li-Cor, 927–85001, Lincoln, NE, USA) and incubated on the membranes for 1 h at RT. Membranes were rinsed 3× 5 min in TBST and 1× 5 min in TBS. All WBs were run in triplicate and averaged for each antibody with all protein signal intensities normalized to total protein content as determined using No-Stain Protein Labeling Reagent (A44717, Invitrogen, Carlsbad, CA). Blots were imaged on the iBright FL 1500 Imaging System (Invitrogen, Carlsbad, CA) and densitometry was performed with IBright Analysis software (Version 5.3.0, Life Technologies-Thermo Fisher Scientific, Waltham, MA).

The following primary antibodies were used: pTau-S202-T205 (AT8) (1:500, MN1020, Thermo Fisher Scientific, Waltham, MA, USA); pTau-T181 (AT270) (1:5000, MN1050, Thermo Fisher Scientific, Waltham, MA, USA); pTau-S202 (1:10000, ab108387, abcam, Waltham, MA, USA); pTau-T217 (1:1000, 44–744, Thermo Fisher Scientific, Waltham, MA, USA); HT7 (1:40000, MN1000, Thermo Fisher Scientific, Waltham, MA, USA); 3R-Tau (1:5000, 05–803, Millipore-Sigma, Burlington, MA, USA); 4R-Tau (1:5000, MABN1185, Millipore-Sigma, Burlington, MA, USA); APP (1:2500, MAB348, Millipore-Sigma, Burlington, MA, USA); NFL (1:1000, MCA-6H112, Encor. Gainesville, FL, USA); MBP (1:10000, ab7349, abcam, Waltham, MA, USA); GFAP (1:40000, NCL-L-GFAP-GA5, Leica, Deer Park, IL, USA); IBA1 (1:2000, ab178847, abcam, Waltham, MA, USA); H2AX (1:1000, 68888–1-Ig, Proteintech, Rosemont, IL, USA).

The following HRP tagged secondary antibodies were used: goat anti-mouse (1:2000, ab97040, abcam, Waltham, MA), goat anti-rabbit (1:2000, ab97080, abcam, Waltham, MA), and goat anti-rat (1:2000, ab97057, abcam, Waltham, MA, USA).

### Immunohistochemistry (IHC):

Each formalin-fixed hemi-brain was cut into a series of 4–5 mm thick coronal slabs and tissue samples were sub-dissected for processing to paraffin blocks using an automated tissue processor (ASP 6025, Leica Biosystems, Nussloch, Germany). Paraffin embedded tissue blocks were cut in a series of 20, 5-μm thick consecutive sections. The first two sections were selected for hematoxylin and eosin (H&E; standard general stain) and Luxol-Fast Blue+PAS (LFB-PAS; myelin stain) stain, while the remaining sections were retained for immunohistochemistry (IHC) procedures. All stained sections were digitally scanned using an Aperio scanner system (Aperio AT2-High Volume, Digital whole slide scanning scanner, Leica Biosystems, Inc., Richmond, IL, USA) and stored in a digital image archive for further assessment and analyses. Preliminary neuropathologic assessments for each section were performed using Aperio ImageScope (Aperio ImageScope, version 2016, Leica Biosystems, Inc., Richmond, IL, USA) to verify presence of immunoreactivity (IR), anatomical localization and neurohistological distribution of each antibody. After the preliminary ImageScope histological inspections (magnification at 20X), a Zeiss Imager A2 (ImagerA2 microscope, Zeiss, Munich, Germany) bright-field microscope with higher magnification lenses (40X, 63X-oil immersion objective) was used to identify and photograph histologic and pathologic details, as needed.

The following IHC antibodies were used: Anti-phosphorylated Tau (pTau) (AT8) (1:200, MN1020, Invitrogen, Thermo-Scientific, Waltham, MA); anti-1–42/1–40 *β*-amyloid (1–42/40 *β*A) (6E10) (1:500, 803001, BioLegend, San Diego, CA); anti-glial fibrillary acidic protein (GFAP) (1:1000, NCL-L-GFAP-GA5, Leica Biosystems, Wetzlar, Germany); anti-ionized calcium-binding adapter molecule 1 (IBA-1) (1:500, 019–19741, FUJIFILM Wako Pure Chemical Corporation, Osaka, Japan); anti-myelin basic protein (MBP) (1:500, ab7349, abcam, Waltham, MA, USA).

### Droplet Digital Polymerase Chain Reaction (dPCR) Procedures:

Due to tissue availability constraints, it was necessary to utilize additional female control samples for dPCR that were not used in WB. Total RNA was extracted from brain tissue samples using the RNeasy Lipid Tissue Mini Kit (Qiagen, Cat. No. 74804, Hilden, Germany). Approximately ≤100 mg of tissue was placed into a 2 mL microcentrifuge tube and homogenized in 1 mL of QIAzol Lysis Reagent with a TissueRuptor. The samples were then incubated at room temperature for 5 minutes. After incubation, 200 μL of chloroform was added, followed by vigorous shaking and another 2–3 min incubation. Phase separation was achieved by centrifuging the samples at 12,000 × g for 15 min at 4 °C. Next, the upper aqueous phase (~600 μL) was transferred to a new tube and mixed 1:1 with 70% ethanol. This mixture was applied to the RNeasy spin column. The column was then washed with Buffers RW1 and RPE before eluting the RNA in 40 μL of RNase-free water. The quantity of the extracted RNA was measured using a NanoDrop (Qiagen Inc). Following RNA extraction, reverse transcription was performed using the SuperScript IV VILO Master Mix (Thermo-Fisher Scientific, Waltham, MA, USA) without ezDNase treatment. The reaction was conducted in a final volume of 20 μL, and the resulting cDNA was stored at −80°C.

Absolute quantification and analysis of cDNA was performed in triplicate using TaqMan gene expression assays (listed below) with droplet digital PCR (dPCR). For dPCR analysis, the cDNA was diluted based on the tissue type. For cortex samples, RT products were diluted at 1:100 for all primers, except for MAPT, where they were diluted to 1:10. Hippocampus and cerebellum RT samples were diluted to 1:500. The dPCR reaction was performed using the QX200 Droplet Digital PCR System (Bio-Rad) and analyzed with QuantaSoft software. Each reaction was prepared by combining 11 μL of dPCR Supermix for Probes (No dUTP), 1 μL of a primer (see gene expression assays below), and 5 μL of nuclease-free water, for a total master mix volume of 17 μL. This master mix was then combined with 5 μL of the diluted cDNA. Following preparation, 20 μL of the final mixture was loaded into a droplet generation cartridge along with 70 μL of droplet generation oil. After droplet generation, 40μL of the droplets were transferred to a 96-well PCR plate, sealed, and amplified in a thermocycler using standard probe cycling conditions. The samples were then read on the QX200 dPCR system, and droplet quality and quantification were assessed using the QuantaSoft Suite (software version 1.7.4.0917).

The following TaqMan gene expression assays (Thermo-Fisher Scientific, Waltham, MA, USA) were used for dPCR: MAPT (Rhesus macaque, assay ID: Rh04269822_m1 – Cat# 4331182), APP (Rhesus macaque, assay ID: Rh01552279_m1 – Cat# 4331182), GFAP (Rhesus macaque, assay ID: Rh00909240_m1 – Cat# 4351372), IBA1 (AIF1) (Rhesus macaque, assay ID: Rh00894882_m1- Cat# 4351372), MBP (Rhesus macaque, assay ID: Rh02797939_m1 - Cat# 4351372) NEFL (gene corresponding to NFL) (Homo sapiens, assay ID: Hs00196245_m1 – Cat# 4331182), and H2AX (histone family member x) (Cynomolgus monkey, Loc 102132314, assay ID: Mf02927011_s1 – Cat# 4351372).

### Statistical analysis:

For each antibody and each examined region, two-tailed unpaired *t*-tests were performed for WB. Two-tailed unpaired *t*-tests were also performed for each of the dPCR targets. Data values reported are mean±standard error of the mean (SEM). Differences with *p*-value ≤ 0.05 were considered significant in all cases. Results were graphed and statistical tests were performed using GraphPad Prism version 10.6.1 for Windows (GraphPad Software, La Jolla, CA).

## Results

### Molecular findings.

**Figure 1:**
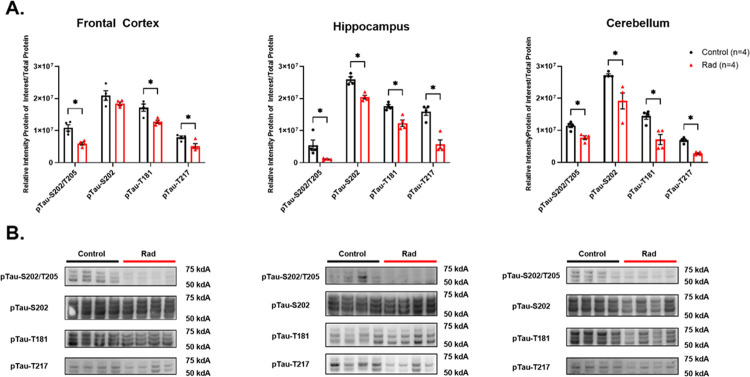
(A.) Western blot (WB) densitometric analysis for the phosphorylated Tau markers: pTau-S202/T205, pTau-S202, pTau-T181, and pTau-T217 in the Frontal Cortex, Hippocampus and Cerebellum from the brains of Control (Unirradiated) (n=4) (black) and Rad (Irradiated) (n=4) (red) rhesus macaques. (B.) Representative WBs for all proteins are shown. Each blot was run in triplicate, and the graphs represent the average of 3 runs. *Indicates *p*<0.05 as determined by unpaired 2-tailed t-test. Error bars represent the standard error of the mean. Original full-length blots are presented in Supplemental Figs. 1–3.

WB quantification analyses revealed a significant reduction in all examined phosphoTau species (pTau-S202-T205, pTau-T181, pTau-T217, and pTau-S202) in RAD vs. CTRL groups across all three brain regions (FCtx, Hippo, and CRB). The only exception was pTau-S202 in the FCtx, which showed a decreasing trend but did not reach statistical significance (Figure 1).

**Figure 2:**
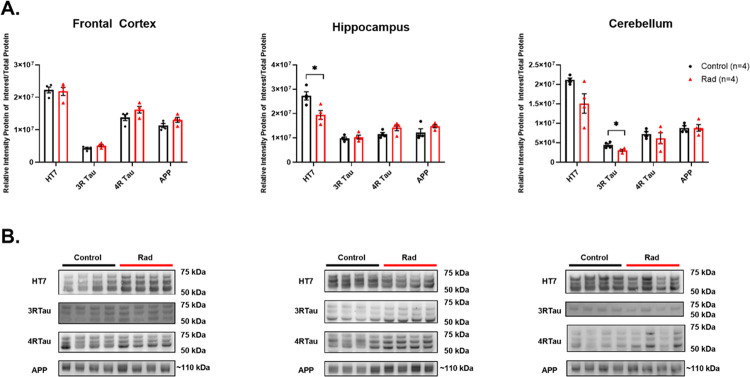
(A.) Western blot (WB) densitometric analysis for the neurodegenerative associated markers: HT7, 3RTau, 4RTau, and APP in the Frontal Cortex, Hippocampus and Cerebellum from the brains of Control (unirradiated) (n=4) (black) and Rad (irradiated) (n=4) (red) rhesus macaques. (B.) Representative WBs for all proteins are shown. Each blot was run in triplicate, and the graphs represent the average of 3 runs. *Indicates *p*<0.05 as determined by unpaired 2-tailed t-test. Error bars represent the standard error of the mean. Original full-length blots are presented in Supplemental Figs. 4–6.

Regarding 3R-Tau, 4R-Tau, and HT7, no significant differences in their expression levels were observed between RAD and CTRL animals across any brain region, except for a notable reduction of 3R-Tau in the CRB and HT7 in the Hippo. Additionally, APP expression remained unchanged across all three regions (Figure 2).

**Figure 3:**
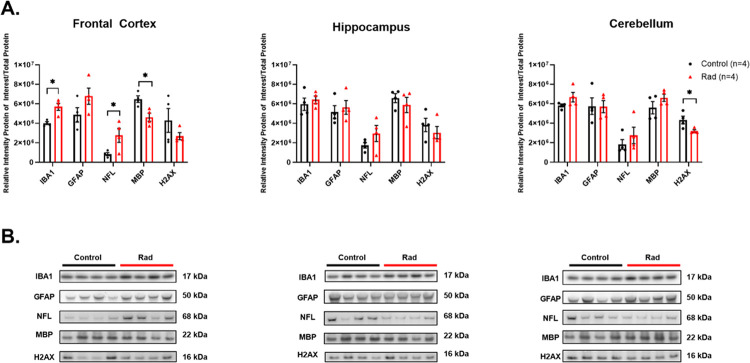
(A.) Western blot (WB) densitometric analysis for the neuroinflammation/neuroplasticity and DNA damage markers: IBA1, GFAP, NFL, MBP, and H2AX in the Frontal Cortex, Hippocampus and Cerebellum from the brains of Control (unirradiated) (n=4) (black) and Rad (irradiated) (n=4) (red) rhesus macaques. (B.) Representative WBs for all proteins are shown. Each blot was run in triplicate, and the graphs represent the average of 3 runs. *Indicates *p*<0.05 as determined by unpaired 2-tailed t-test. Error bars represent the standard error of the mean. Original full-length blots are presented in Supplemental Figs. 7–9.

In terms of neuroinflammation/neuroplasticity markers, GFAP expression (indicative of astrocyte activation) was not significantly changed across all three regions, while IBA1 expression (indicative of microglial activation) was increased only in the FCtx of RAD vs. CTRL animals. Lastly, NFL demonstrated an increase in the FCtx of RAD vs. CTRL animals with a similar trend in Hippo and CRB, whereas MBP levels were decreased in the FCtx of RAD vs CTRL animals but remained unchanged in Hippo and CRB. H2AX demonstrated a significant decrease in the CRB of RAD vs. CTRL animals with a similar trend in FCtx and Hippo (Figure 3).

**Figure 4:**
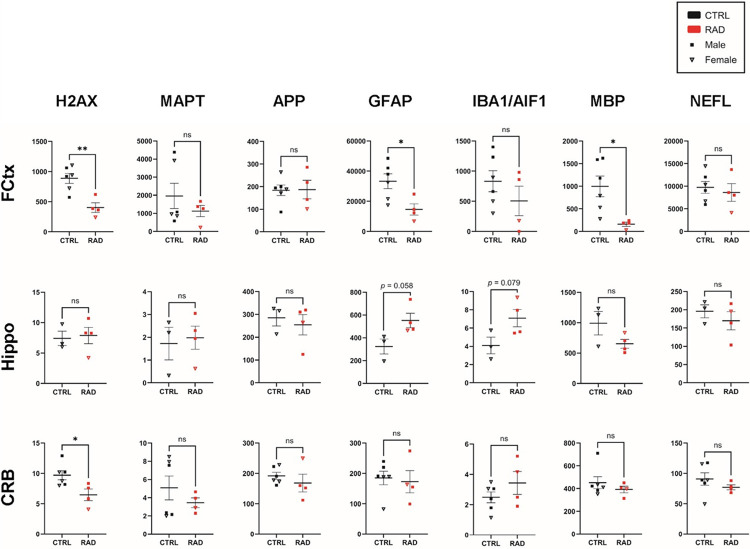
dPCR identified significant decreases in the FCtx of GFAP and MBP gene expression, as well as decreased H2AX expression in both the FCtx and CRB. There were no significant changes in measured gene expression in Hippo. Male vs female comparisons and graphs including only the samples used in WB are presented in Supplemental Fig. 10.

In the FCtx, dPCR identified a significant decrease in the absolute gene expression of H2AX (*p* = 0.004), GFAP (*p* = 0.025) and MBP (*p* = 0.019) following 5.8 Gy irradiation (Figure 4). MAPT expression was not significantly different between RAD (n=4) and CTRL animals (n=6), possibly due to the limited sample sizes. It should be noted that comparing only those animals with corresponding WB data shows a significant reduction in MAPT expression among RAD animals, see Supplemental Fig. 10). APP and IBA1 expression were also unaffected by irradiation. We did assess possible sex differences in the control group (n=3 males vs. n=3 females) and identified significantly reduced expression of GFAP and MBP among females, although the trend for the decreasing effect of radiation was still even more pronounced.

In the Hippo there were no significant changes identified by dPCR for any targets. However, it should be noted that the CTRL sample size was smaller (n = 3) for this set of analyses due to limited tissue availability. Thus, we would draw attention to the trending increase in GFAP (*p* = 0.058) and IBA1 (*p* = 0.079), which may warrant further investigation in the future.

In the CRB, dPCR again identifies a significant decrease in H2AX expression. No other targets appear altered in this region by 5.8 Gy irradiation.

### Neuropathological findings.

**Figure 5:**
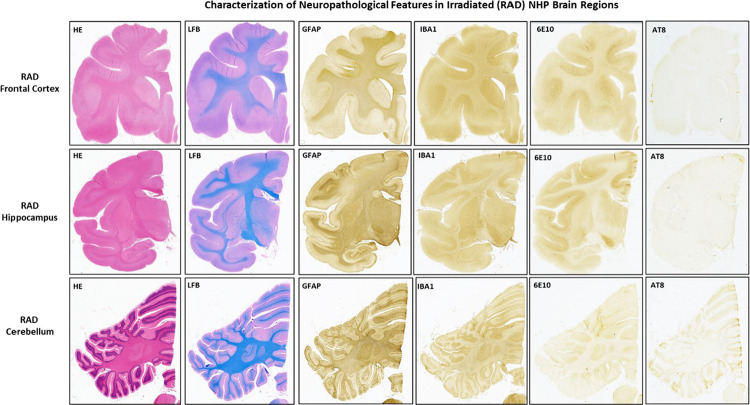
Systematic neuropathological assessment of brain tissue from irradiated (Macaca mulatta) animals (RAD). Representative images from the Frontal Cortex (FCtx), Hippocampus (Hippo), and Cerebellum (CRB) stained with standard histological and immunohistochemical (IHC) markers. Hematoxylin and Eosin (H&E) and Luxol-Fast Blue (LFB) staining show general morphology, cytology, and myelination, did not reveal overt signs of neurodegeneration, neuronal loss, vascular pathology or myelin pallor in the Frontal Cortex, Hippocampus and Cerebellum. Immunohistochemistry (IHC) for Glial Fibrillary Acidic Protein (GFAP), an astrocyte activation marker, shows basal, non-reactive morphology across all three regions examined. IHC for Ionized Calcium-Binding Adapter Molecule 1 (IBA1), a microglial marker, displays quiescent or resting microglial morphology in the FCtx. Equivalent staining for H&E, GFAP, and IBA1 are shown for the Hippocampus and for the Cerebellum (CRB). Globally, the qualitative assessment across all regions and markers showed no evidence of widespread reactive astrogliosis or microgliosis at the time of euthanasia. No extracellular *β*-amyloid deposits (6E10) or phosphorylated-Tau (AT8) accumulations were detected across any regions assessed. Original magnification is 20x.

**Figure 6:**
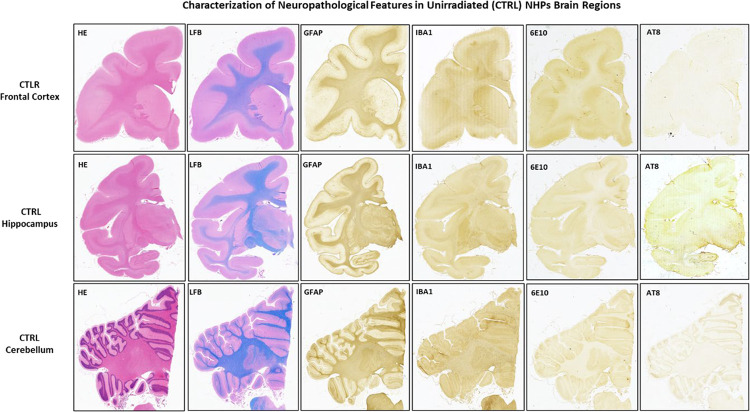
Systematic neuropathological assessment of brain tissue from unirradiated (Macaca mulatta) animals (Control). Representative images from the Frontal Cortex (FCtx), Hippocampus (Hippo), and Cerebellum (CRB) stained with standard histological and immunohistochemical (IHC) markers. Hematoxylin and Eosin (H&E) staining shows normal cellular and tissue architecture in the FCtx. IHC for Glial Fibrillary Acidic Protein (GFAP) demonstrates normal, non-reactive astrocytic morphology in the FCtx. IHC for Ionized Calcium-Binding Adapter Molecule 1 (IBA1) shows the typical ramified, non-reactive morphology of resting microglia in the FCtx. Equivalent staining for H&E, LFB, GFAP, and IBA1 are shown for the Hippocampus and Cerebellum. These images serve as the baseline control for comparison with the irradiated group (Figure 5). No *β*-amyloid (6E10) or tau (Tau) lesions were detected across all three regions examined. Original magnification is 20x.

**Figure 7:**
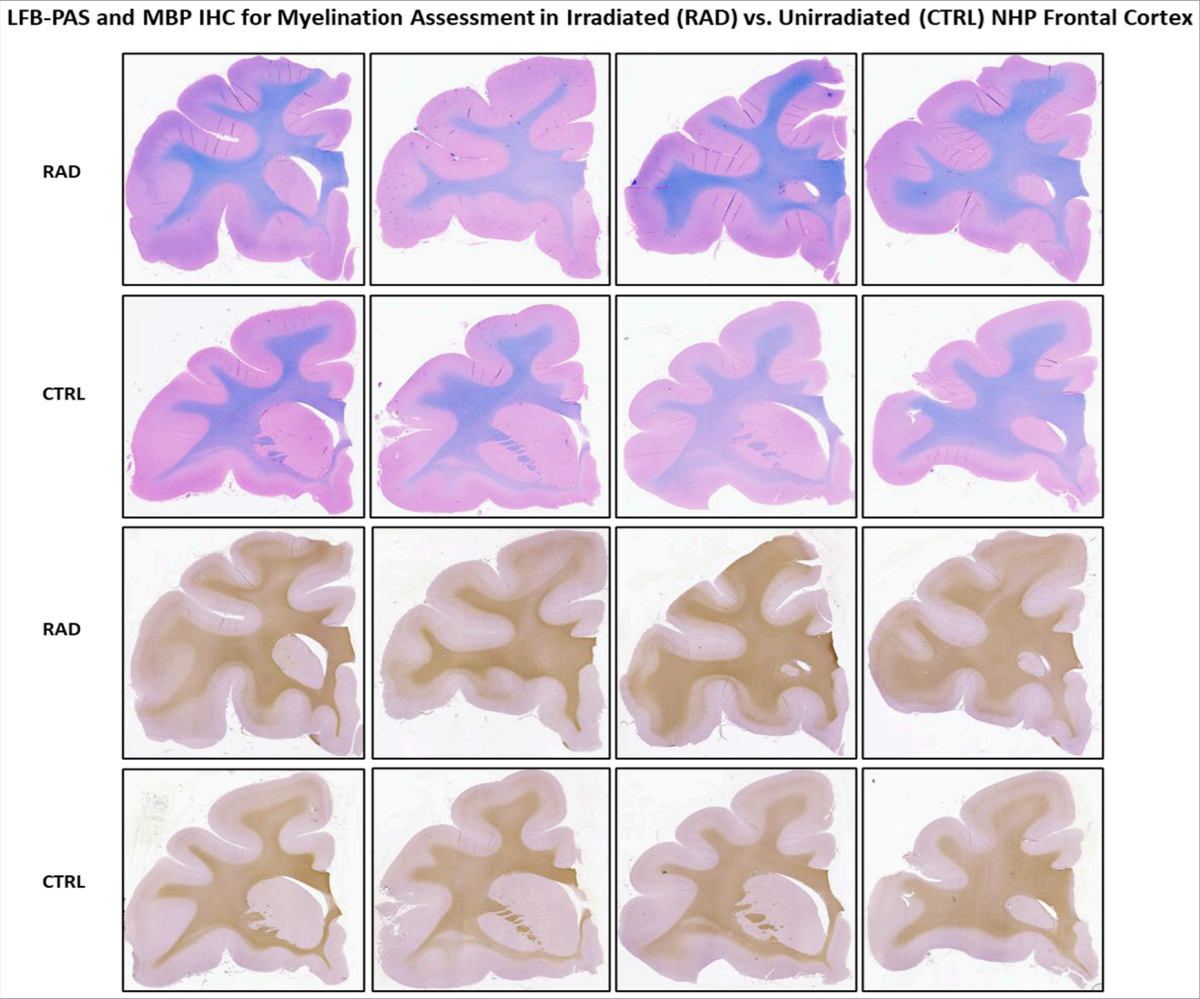
Qualitative assessment of myelination using Luxol Fast Blue (LFB) and Myelin Basic Protein (MBP) immunohistochemistry in the Frontal Cortex (FCtx). Representative images of myelin staining in the FCtx of irradiated (RAD) and unirradiated (CTRL) rhesus macaques. Luxol Fast Blue-PAS (LFB+PAS) staining of the FCtx shows no overt qualitative difference in myelin density or structural integrity between RAD and CTRL animals. IHC for Myelin Basic Protein (MBP), a major component of myelin, also reveals no pronounced qualitative reduction in immunoreactivity between the RAD and CTRL FCtx. Despite quantitative Western Blot (WB) and dPCR analyses showing significantly reduced MBP protein and gene expression in the FCtx of RAD animals, the corresponding LFB-PAS and MBP IHC qualitative assessments did not detect major gross changes in myelination levels. Original magnification is 20x.

The systematic neuropathological assessments performed by histology and IHC staining revealed no overt signs of neurodegeneration, morphometric abnormalities, or vascular/perivascular lesions in any of the examined regions. IHC assessment of GFAP and IBA1 markers across those three regions of the brain showed no major evidence of a diffused increased level of neuroinflammation, such as astrogliosis, or widespread reactive microgliosis (Figures 5 and 6). Moreover, LFB-PAS and MBP stain did now show qualitative changes in terms of myelination levels between RAD vs. CTRL brains, including the FCtx (Figure 7), a region of the brain region where WB quantitative analyses detected lower levels of MBP in that same region of the brain (see Discussion).

## Discussion

To the best of our knowledge, this is the first investigation to report a marked short-term reduction of different forms of pTau levels in the brains of NHPs following a single acute total-body γ-irradiation with a 5.8Gy dose (~LD_30_/_60_). Our quantitative molecular analyses - conducted either on the day of mortality (CTRL) or approximately two months post-exposure (RAD) - revealed a significant decrease in all examined pTau species (pTau-S202-T205, pTau-T181, pTau-T217, and pTau-S202) across three distinct brain regions: FCtx, Hippo, and CRB. These brain areas are each characterized by unique neurohistological architectures and functions, suggesting a global effect of γ-irradiation on Tau phosphorylation. These observations, consistent with previous findings in another large animal model (minipig) [22,23], strongly support the hypothesis that Tau phosphorylation is radiosensitive in primates and may represent a general neurobiological phenomenon in large mammalian systems.

The observed reduction of pTau levels, particularly in the Hippo, may hold therapeutic potential for AD and other tauopathies. NFT formation and accumulation, driven by hyperphosphorylated Tau, is one of the primary pathological hallmarks of AD [34]. In particular, the burden of NFTs in regions like the Hippo correlates tightly with cognitive decline [39–43]. Theoretically, by refining focal and modulated irradiation procedures, targeted protocols could be developed to establish a radiotherapeutic strategy that reduces pathological pTau species in critical brain regions. While the exact subcellular localization and molecular mechanisms underlying these pTau reductions require further investigation, our findings suggest that the assessed pTau species are radiosensitive at the 5.8 Gy dose level. This aligns with previous findings in a minipig model, supporting pTau radiosensitivity as a general phenomenon in large animals. Globally, these results may open new avenues for studying neurobiological-irradiation interactions and developing novel therapeutic approaches, including focal radiation-based interventions.

While the specific Tau-radiosensitivity related mechanisms remain to be fully elucidated, the almost global reduction across various forms of pTau (Figure 1), without a corresponding significant reduction in total Tau (HT7, 3R-Tau, 4R-Tau) protein expressions in most regions (Figure 2), suggests that γ-radiation may not be primarily acting by inducing general protein catabolism or inhibiting Tau gene transcription (MAPT dPCR was indeed non-significant (Figure 4). Instead, the radiation insult may activate pTau-specific phosphatases (e.g., PP2A, PP1, PP5) either directly or via upstream signaling cascades (e.g., calcium signaling, oxidative stress pathways). Another possible mechanism could be the inhibition of pTau-specific kinases (e.g., GSK-3β, CDK5). Also, the possible enhancement of clearance mechanisms by microglia or astrocytes, which selectively phagocytose pTau before the formation of insoluble NFTs could represent another not necessarily mutually exclusive mechanism to explain these radiation-induced pTau reduction phenomena (44,45).

In contrast to the potentially beneficial effects on pTau, our findings also highlight possible radiation-induced neurotoxicity effects, particularly in the FCtx. We observed a significant increase in NFL, a well-established biomarker of axonal damage, and a concurrent decrease in MBP, indicating demyelination. These results, with the MBP reduction confirmed at both the protein and transcriptional levels, strongly suggest that γ-irradiation at this dose inflicts direct structural damage to neurons and their protective myelin sheaths. This duality - the reduction of a pathological protein accompanied by signs of neuronal injury - presents a critical challenge. It underscores that any potential therapeutic application of radiation for neurodegenerative diseases must carefully balance intended benefits with unintended neurotoxic side effects, necessitating comprehensive dose-response studies. Reports of radiation-induced cognitive dysfunction in NHPs at long-term recovery timepoints (7+ years post exposure) following higher levels of irradiation (6.5–8.05 Gy) supports the potential for neurotoxic effects (46). This duality - the reduction of a pathological protein accompanied by signs of neuronal injury - presents the central challenge for developing radiation-based therapies for tauopathies, and likely for other proteinopathy. Similarly to pharmacological treatments, any potential therapeutic protocol must rigorously determine the therapeutic window where the Tau-lowering effect (beneficial) is achieved at a dose and dose rate that minimizes axonal/myelin injury (detrimental). Indeed, other studies of NHPs have reported evidence of white matter injury as long-term outcomes of irradiation exposure (47,48). Our use of a total-body LD30/60 dose (5.8 Gy), while providing a critical reference point for radiation pathology, clearly resulted in an unacceptable level of neurotoxicity. Future work then must focus on focal brain irradiation at lower doses to decouple these two opposite effects.

Our neuroinflammatory assessment revealed a complex, region-specific, glial response. While we did not observe a significant increase in GFAP, suggesting the absence of pronounced astrogliosis, we did identify a localized increase in microglial reactivity (IBA1) in the FCtx. This could represent a pro-inflammatory response to the neuronal damage indicated by the NFL and MBP changes. However, generally, microglial activation is not exclusively detrimental; it may also reflect a compensatory, neuroprotective response aimed at clearing cellular debris and pathological proteins like Tau (49). The trending increase in GFAP and IBA1 gene expression in the Hippo, though not statistically significant, warrants further investigation with larger sample sizes. More specifically, the inconsistency between molecular and neuropathological data requires careful consideration.

The WB/dPCR results (quantitative molecular changes) are likely detecting subtle, early-stage, or localized cellular reactions that precede the morphological changes visible via IHC (qualitative, histological). The increase in microglial protein (IBA1) in the FCtx may represent an early, localized phagocytic attempt to clear damaged myelin/axonal debris (50–52). The lack of overt astrogliosis (GFAP) in IHC, coupled with the reduction in GFAP gene expression in FCtx, is particularly notable and contrasts with typical chronic CNS injury models.

Furthermore, our data indicate that γ-irradiation does not significantly alter amyloid precursor protein (APP) expression at the protein or transcriptional level. While not directly assessed, this suggests that γ-irradiation does not promote the production of its cleavage product, *β*-amyloid, a key driver of neuritic plaque formation in AD. Future amyloid-specific investigations are necessary to confirm this hypothesis. It has been reported that higher levels of radiation can induce changes in ApoE in a region dependent manner in NHPs long after exposure, so it is crucial that these pathological outcomes are fully explored (53). Finally, the observed decrease in H2AX gene expression in the FCtx and CRB may represent a compensatory mechanism to counteract acute increases in its phosphorylated form (γH2AX), a marker of DNA damage that recruits repair machinery (54). In fact, the significant decrease in H2AX gene expression in the FCtx and CRB (Figure 4) can be interpreted as a potential compensatory counter-response to the expected acute increase in the DNA double-strand break marker such as γH2AX, which was not detectable in our samples (data not shown). Since γ-radiation is a potent inducer of DNA damage, the initial injury would cause a massive and immediate increase in γH2AX (phosphorylated-H2AX protein). The observed decrease in H2AX mRNA in the acute recovery phase (a few weeks post-exposure) could well reflect a feedback mechanism where the cell downregulates the transcription of the parent histone to mitigate the overwhelming acute signal or as part of the triage/repair process. Future studies should quantify the γH2AX protein to confirm the initial DNA damage and this proposed compensatory mechanism. It is important to acknowledge the limitations of this study. The NHPs were relatively young, non-transgenic, and free from pre-existing neuropathology. Whether the Tau-lowering effect of γ-irradiation extends to aged NHPs or NHP models of AD remains to be determined. The study’s statistical power was also limited, and future work would benefit from a formal power analysis to ensure adequate sample sizes. Additional studies will be crucial to assess the short- and long-term effects of different radiation doses, dose rates, and exposure modalities (e.g., total-body versus focal brain irradiation) on these complex neurobiological pathways. We would like to emphasize though that the use of the rhesus macaque NHP model seems to provide unprecedented translational relevance, particularly for brain studies. However, the use of a lethal-range total-body dose (LD_30/60_) led to the early, moribund-driven euthanasia of two animals. Consequently, the observed molecular changes represent the effects of acute, severe radiation sickness with neurological components, rather than chronic or purely neurodegenerative effects. The varying post-exposure survival times (ranging from early mortality to ~60 days) contribute to the heterogeneity of the findings and must be considered when interpreting the ‘time-point’ of the results. The finding that APP protein and gene expression remained unchanged (Figure 2) is also significant. It suggests that, in this acute setting, γ-irradiation at this dose does not immediately drive the amyloid pathway, a key upstream pathological hallmark of AD. This partially separates the radiation-induced Tau response from the typical AD cascade and supports a more direct or amyloid-independent mechanism for the pTau reduction. Future efforts should be directed at beta-amyloid protein detection (using IHC antibodies like 6E10) to conclusively rule out acute amyloidopathy.

While the observed reduction in multiple soluble pTau species following 5.8 Gy γ-radiation is a potentially paradigm-shifting finding, several factors regarding the study design and the nature of the radiation exposure warrant careful consideration. In fact, we acknowledge that the 5.8 Gy total-body irradiation (TBI) utilized in this study falls within the LD50/60 range for Rhesus macaques, as evidenced by the 50% lethality rate observed prior to the 60-day endpoint. Also, the concomitant increase in NFL levels suggests that this pTau reduction occurred alongside acute neuronal injury. These findings indicate that while a high-dose, acute systemic challenge can trigger tau clearance, the current protocol exceeds the therapeutic window for clinical application in AD patients. However, future studies must explore the “radiation-hormesis” hypothesis using significantly lower, non-lethal doses or localized, fractionated cranial-radiotherapy protocols to decouple the beneficial protein-clearing effects from neurotoxic damage.

Moreover, although our results characterize the regional impact of radiation on pTau, the precise molecular mechanisms remain to be fully elucidated. We speculate that it is possible that radiation-induced pTau reduction is mediated by the activation of the autophagy-lysosomal pathway or altered proteasomal activity; though, further mechanistic investigations are required to confirm these pathways. We also acknowledge the limitations inherent in the small sample size and the use of healthy, rather than AD-model, primates. While AD models are essential for testing efficacy against established pathology, utilizing healthy NHPs was a necessary first step to determine how radiation modulates physiological tau metabolism in a brain that closely mirrors human neuroanatomy. It is also important to emphasize that all specimens were processed using equivalent post-mortem intervals (PMIs) and standardized fixation and freezing protocols to ensure that the observed differences were not artifacts of post-mortem degradation.

## Conclusion

This first-in-NHP study provides crucial evidence that acute total-body γ-irradiation (5.8 Gy) induces a profound, region-wide reduction in pathological pTau species, validating a radiosensitivity phenomenon observed in other large animals. Simultaneously, this dose elicits region-specific neurotoxicity characterized by axonal damage (NFL increase) and demyelination (MBP decrease), particularly in the FCtx.

This work establishes that the NHP model may be a critical platform for studying radiation-neurobiology and underscores the need for highly refined, low-dose, and focal brain irradiation studies to ethically and safely explore the potential of radiation as a novel therapeutic strategy for Tau-driven neurodegenerative diseases, such as AD. The complex and sometimes contradictory glial and DNA-damage responses highlight the need for larger cohort studies that include a full dose-response curve and chronic follow-up time points.

## Supplementary Material

Supplementary Files

This is a list of supplementary files associated with this preprint. Click to download.

- SupplementaryInformation.pdf

## Data Availability

The datasets used and/or analyzed during the current study and supporting the conclusions of this article are included in this article and supplementary materials provided. These datasets are also available from the corresponding author on request.
